# Heart Rate, Electrocardiographic Subclinical Myocardial Injury, and Long-Term Mortality

**DOI:** 10.3390/jcm15124405

**Published:** 2026-06-06

**Authors:** Patrick Cheon, Mohamed A. Mostafa, Mai Z. Soliman, Richard Kazibwe, Elsayed Z. Soliman

**Affiliations:** 1 Winston-Salem Campus, Wake Forest University School of Medicine, Winston Salem, NC 27157, USA; patrick.cheon@wfusm.edu; 2 Epidemiological Cardiology Research Center (EPICARE), Department of Cardiovascular Medicine, Wake Forest University School of Medicine, Medical Center Blvd, Winston Salem, NC 27157, USA; moh.adel.mostafa@gmail.com (M.A.M.); mai.soliman@wfusm.edu (M.Z.S.); 3 Department of Medicine, Wake Forest University School of Medicine, Winston Salem, NC 27157, USA; richard.kazibwe@wfusm.edu

**Keywords:** heart rate, subclinical myocardial injury, cardiovascular mortality, NHANES, preventive cardiology

## Abstract

**Objective:** Although an elevated resting heart rate is linked to higher mortality, the pathways underlying this relationship are not fully defined. One candidate mechanism is subclinical myocardial injury (SCMI), an asymptomatic form of cardiac damage indexed by a Cardiac Infarction/Injury Score (CIIS) of at least 10, which has prognostic value for adverse cardiovascular (CV) outcomes. **Methods:** We analyzed 7152 participants from NHANES III who underwent electrocardiogram (ECG) recording and were free of CV disease. Heart rate was categorized as bradycardia (≤50 bpm), normal (>50–<100 bpm), or tachycardia (≥100 bpm). Mortality was assessed through National Death Index linkage. Logistic and Cox regression models evaluated associations with SCMI and mortality, respectively, and attenuation was assessed by change in hazard ratios after adjusting for SCMI. **Results:** SCMI was present in 1744 (24.3%) participants. Tachycardia was associated with increased odds of SCMI (adjusted OR 2.34, 95% CI 1.42–3.88). Over 13.9 years median follow-up, 2311 (32.3%) died from all causes and 933 (13.1%) from CV causes. Tachycardia was associated with increased all-cause mortality (HR 3.58, 95% CI 2.63–4.88) and CV mortality (HR 2.05, 95% CI 1.06–3.79). Adjustment for SCMI attenuated the tachycardia–CV mortality association by 8.6% and all-cause mortality by 5%. Bradycardia was not associated with SCMI or mortality. **Conclusions:** These findings suggest that SCMI modestly attenuates the heart rate–mortality association, suggesting that silent myocardial damage may represent one potential intermediary pathway.

## 1. Introduction

Elevated resting heart rate is associated with increased risk of all-cause and cardiovascular (CV) mortality across diverse populations [1,2,3,4]. This association persists after adjustment for traditional CV risk factors, suggesting that elevated heart rate may contribute to underlying pathophysiological processes beyond its role as a simple vital sign. Proposed mechanisms include increased myocardial oxygen demand, reduced diastolic perfusion time, and autonomic imbalance [5,6]. However, these mechanisms do not fully account for the excess mortality risk associated with elevated heart rate, and the precise pathways linking elevated heart rate to adverse outcomes remain incompletely understood.

Subclinical myocardial injury (SCMI) refers to myocardial damage that develops without accompanying clinical manifestations. It is quantifiable through the Cardiac Infarction/Injury Score (CIIS), a validated electrocardiogram (ECG) derived metric, and prior cohorts have linked higher scores to elevated risk of subsequent CV events and death [7,8,9]. Given that elevated resting heart rate increases myocardial oxygen demand, SCMI may represent a plausible mechanistic pathway through which elevated heart rate contributes to mortality [5,6]. However, the relationship between resting heart rate and SCMI has not been systematically evaluated, and whether SCMI partially accounts for the association between elevated heart rate and mortality remains unknown.

We hypothesized that a higher resting heart rate would correspond to a greater burden of SCMI, and that accounting for SCMI would partly attenuate the association between resting heart rate and long-term all-cause and CV mortality. Utilizing the Third National Health and Nutrition Examination Survey (NHANES III) data, we examined the cross-sectional relationship between resting heart rate and SCMI and evaluated whether SCMI partially explains the association between elevated heart rate and long-term all-cause and CV mortality.

## 2. Methods

### 2.1. Study Population

Conducted from 1988 to 1994 by the NCHS, a division of the Centers for Disease Control and Prevention, NHANES III was a national survey evaluating the health and nutritional status of community-dwelling children and adults across the United States. Written informed consent was obtained from every participant, and the NCHS institutional review board approved the study protocol. Detailed accounts of the survey design, methods, and data access have been published previously [10].

**Figure 1:**
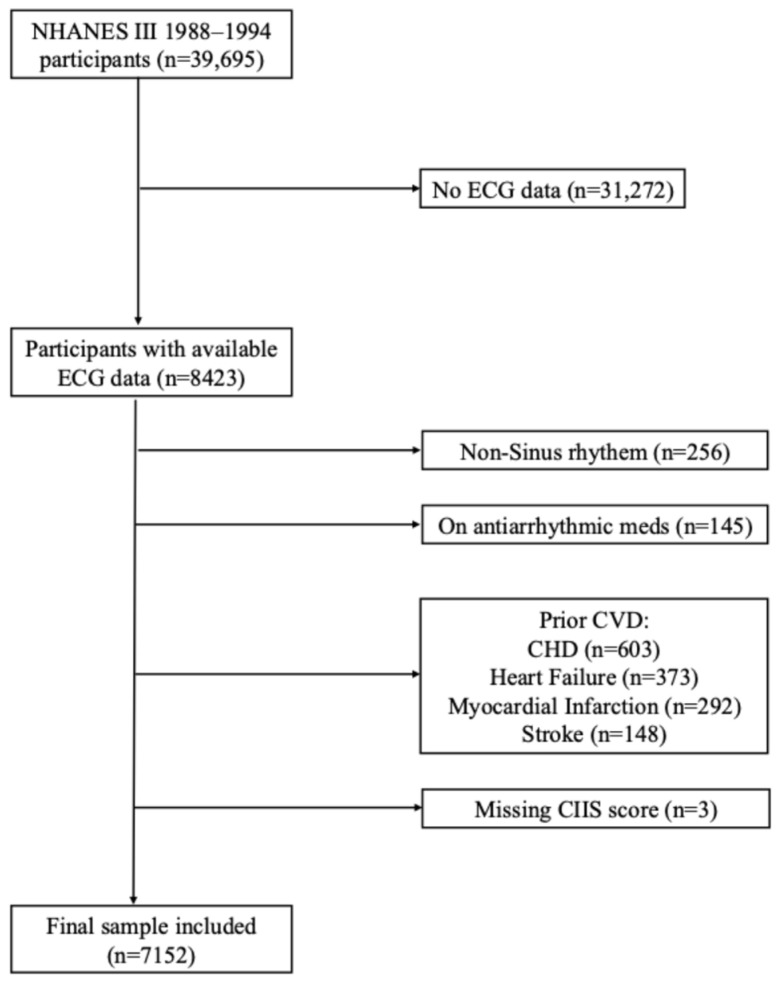
Study flowchart beginning with 39,695 National Health and Nutrition Examination Survey Third phase participants. After excluding 31,272 without electrocardiogram data, 8423 remained. Sequential exclusions for non-sinus rhythm, antiarrhythmic medication use, prior cardiovascular disease, and missing cardiac infarction injury score data resulted in a final analytic sample of 7152 participants. Abbreviations: ECG, electrocardiogram; CVD, cardiovascular disease; CHD, coronary heart disease; CIIS, cardiac infarction/injury score.

The present analysis was restricted to participants with a baseline ECG, which under the survey design was obtained only in those aged 40 years and older. We then removed individuals with established CVD (prior myocardial infarction, heart failure, coronary heart disease, or stroke), those who were not in sinus rhythm, those receiving antiarrhythmic therapy, and those missing variables required for the analysis. These exclusions yielded a final analytic cohort of 7152 participants (Figure 1).

### 2.2. Ascertainment of Resting Heart Rate

Resting 12-lead ECGs were recorded with a Marquette MAC 12 electrocardiograph (Marquette Medical Systems, Milwaukee, WI, USA) as part of the physical examination performed at the NHANES III mobile examination center. Resting heart rate was derived from the standard 12-lead ECG recording. For the primary analysis, participants were categorized into three groups based on resting heart rate: bradycardia (≤50 beats per minute [bpm]), normal heart rate (>50 to <100 bpm), and tachycardia (≥100 bpm). In secondary analyses, heart rate was also modeled as a continuous variable per 10 bpm increase.

### 2.3. Defining Subclinical Myocardial Injury (SCMI)

SCMI was ascertained from the ECG-based CIIS. Digitized ECG signals were transmitted for centralized analysis to the Epidemiological Cardiology Research Center (EPICARE) at Wake Forest University School of Medicine (Winston-Salem, NC, USA). Trained technicians first reviewed each tracing visually, after which the recordings were processed automatically through the GE 12-SL program (Marquette Medical Systems, Milwaukee, WI, USA).

The CIIS applies a weighted algorithm to quantitative ECG parameters to estimate the probability of myocardial injury or ischemia. Its composite value draws on 15 components in all, consisting of 11 categorical and 4 continuous variables measured from the standard 12-lead ECG. Specifically, the score integrates Q-wave duration and amplitude, R-wave amplitudes, ST-segment deviations, and T-wave abnormalities evaluated across their spatial distribution in multiple leads, with each component assigned a weighted coefficient derived from prior validation cohorts comparing ECG tracings of patients with angiographically confirmed myocardial infarction to those of healthy controls. Unlike conventional Minnesota Code criteria for prior myocardial infarction, which rely on dichotomous thresholds for individual waveform abnormalities, the CIIS provides a continuous, probabilistic estimate of myocardial injury burden by combining multiple graded ECG features into a single composite score. As such, the CIIS captures a spectrum of myocardial pathology, including ischemia, subclinical infarction, and fibrosis, rather than solely identifying discrete infarction patterns [7,8].

Within the NHANES III database, CIIS values had been scaled up by a factor of 10 at entry so that decimals could be avoided; we reversed this by dividing each value by 10 to recover the native scale before analysis. SCMI was then defined as a CIIS of at least 10, consistent with cutoffs applied in earlier studies [11,12,13,14,15].

### 2.4. Ascertainment of Mortality

All-cause and CV mortality served as the primary endpoints and were captured by linking participants to the National Death Index, with follow-up extending to 31 December 2015. Deaths attributed to CV causes were classified according to the underlying-cause-of-death codes from the Ninth and Tenth Revisions of the International Classification of Diseases (ICD-9 and ICD-10).

### 2.5. Other Variables

Participant demographics (age, sex, race), current or former smoking, educational attainment, family income, and medication use were obtained by self-report during an in-home interview. Body mass index (BMI), derived from height and weight recorded at the mobile examination center, was expressed as kilograms per square meter (kg/m^2^). Seated systolic and diastolic blood pressures were taken up to three times and averaged. Hypertension was defined by a systolic pressure of 140 mmHg or higher, a diastolic pressure of 90 mmHg or higher, or treatment with antihypertensive agents. Diabetes mellitus was identified by a fasting glucose of 126 mg/dL or greater or use of glucose-lowering therapy, and dyslipidemia by total cholesterol level or use of lipid-lowering medication. A physician-reported diagnosis served to establish thyroid disease. Total cholesterol, serum creatinine, glucose, and the remaining metabolic panel analytes were quantified by laboratory methods documented by the National Center for Health Statistics.

### 2.6. Statistical Analysis

Participant characteristics were examined by SCMI status, with categorical variables assessed using Chi-square tests. For continuous variables, distributional assumptions were verified in advance through Q–Q plots and formal normality testing. Variables meeting normality are reported as means ± SD and compared by independent samples t-tests, whereas skewed variables are reported as medians with IQR and compared by Wilcoxon rank-sum tests. Categorical data are summarized as counts and percentages. The cross-sectional relationship between resting heart rate and SCMI was evaluated with multivariable logistic regression. Heart rate entered the models in two forms, as a category (bradycardia, normal, tachycardia) and as a continuous term scaled per 10 bpm increment. Model 1 included sociodemographic covariates: age, sex, race/ethnicity, years of education, and family income. Model 2 added CV risk factors, namely smoking, alcohol use, antihypertensive therapy, body mass index, systolic blood pressure, serum creatinine, dyslipidemia, diabetes, and thyroid disease.

Associations of resting heart rate with all-cause and CV mortality were assessed using multivariable Cox proportional hazards models. Model 1 accounted for the sociodemographic covariates above, and Model 2 additionally incorporated the CV risk factors specified previously. Model 3 was additionally adjusted for the presence of SCMI to evaluate whether SCMI attenuates the association between heart rate and mortality. The proportional hazards assumption was evaluated using Schoenfeld residuals and visual inspection of log-log survival plots; no significant violations were identified.

To assess the potential explanatory role of SCMI in the relationship between resting heart rate and mortality, we calculated the percentage change in hazard ratios (HR) after adding SCMI to the fully adjusted models using the formula: (HR_Model2 − HR_Model3) / (HR_Model2 − 1) × 100. This approach quantifies the proportion of the excess risk associated with elevated heart rate that is explained by SCMI [16,17].

To examine whether the resting heart rate (per 10 bpm increase)–SCMI association held across clinically relevant strata, we conducted subgroup analyses by age (≤65 vs. >65 years), sex, race/ethnicity, hypertension, diabetes, and BMI category (normal, overweight, obese). Effect modification was assessed formally by adding interaction terms to the fully adjusted models.

All analyses were conducted without incorporating NHANES survey weights; therefore, the reported associations are valid for the analytic sample but should not be interpreted as nationally representative estimates of the U.S. population. Prior methodological work indicates that unweighted regression analyses yield unbiased and often more efficient estimates of exposure–outcome associations when key sampling-related covariates are included in the model [18,19,20]. This analytic strategy aligns with approaches used in several previous NHANES III investigations [21,22]. All statistical analyses were performed using The Jamovi project (2025) (Version 2.4.14.0), retrieved from https://www.jamovi.org. A two-sided *p*-value of 0.05 was used for hypothesis testing.

## 3. Results

### 3.1. Baseline Characteristics

The study included 7152 participants with a mean age of 59.0 ± 13.3 years; 53.4% were women, and 72% were non-Hispanic whites. The mean resting heart rate was 68.5 bpm; 95.7% of participants had a normal heart rate, 3.3% had bradycardia, and 1.0% had tachycardia. SCMI was present in 1744 participants (24.3%). As compared with participants without SCMI, those with SCMI were older, more likely to be male, and more likely to have diabetes, hypertension, or current smoking. The prevalence of SCMI varied according to heart rate category: 45.1% among participants with tachycardia, 24.4% among those with normal heart rate, and 18.9% among those with bradycardia (Table 1).

### 3.2. Association Between Resting Heart Rate and SCMI

Tachycardia was associated with more than twice the odds of SCMI as compared with normal heart rate, an association that persisted after adjustment for CV risk factors. Conversely, bradycardia appeared protective against SCMI. The graded nature of this relationship was confirmed when heart rate was modeled continuously: each increase of 10 bpm was associated with a 14% increase in the odds of SCMI, independent of traditional risk factors (Table 2).

### 3.3. Resting Heart Rate and Mortality Risk

**Figure 2:**
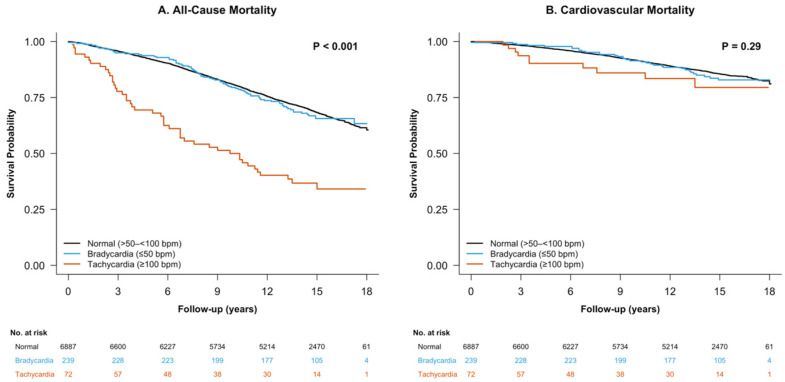
Kaplan–Meier survival curves stratified by resting heart rate categories. Panel (**A**) presents all-cause mortality and Panel (**B**) presents cardiovascular mortality across three resting heart rate groups: normal (>50–<100 bpm), bradycardia (≤50 bpm), and tachycardia (≥100 bpm). Abbreviation: bpm, beats per minute. Kaplan–Meier survival curves over 18 years stratified by heart rate category. For all-cause mortality, participants with tachycardia (≥100 beats/min) had significantly lower survival compared with those with normal heart rates, with an estimated 18-year survival probability of approximately 35% versus 62%, respectively. Cardiovascular mortality curves show no significant difference across the three groups (*p* = 0.29).

During a median follow-up of 13.9 years, 2311 participants (32.3%) died, including 933 (13.1%) from CV causes. In continuous analyses, each 10 bpm increment in resting heart rate was independently associated with higher all-cause mortality (HR 1.20, 95% CI 1.16–1.24) and CV mortality (HR 1.16, 95% CI 1.10–1.23) after full adjustment, confirming a graded dose–response relationship. In categorical analyses, tachycardia was associated with more than three times the risk of all-cause death (HR 3.58, 95% CI 2.63–4.88) and twice the risk of CV death (HR 2.05, 95% CI 1.06–3.79) compared with normal heart rate. Among the 71 participants with tachycardia, 46 (64.8%) died from all causes and 10 (14.1%) from CV causes during follow-up. In contrast, bradycardia showed no adverse association with mortality. Of note, unadjusted Kaplan–Meier curves for CV mortality did not reach statistical significance across heart rate groups (log-rank *p* = 0.29), likely reflecting both the relatively small tachycardia subgroup with limited event counts and confounding by age and comorbidity burden that obscured the underlying association. The independent relationship between tachycardia and CV mortality emerged after multivariable adjustment, consistent with a suppression effect in which removal of negative confounding unmasks the true association (Table 3, Figure 2).

### 3.4. Attenuation by SCMI

**Figure 3:**
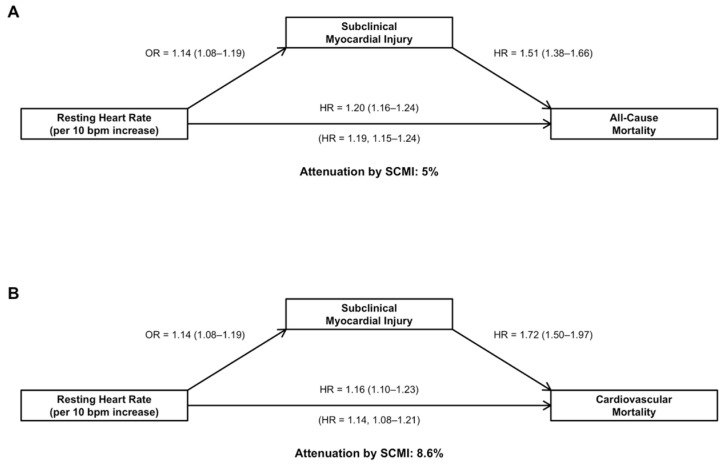
Attenuation of the association between resting heart rate and mortality after adjustment for subclinical myocardial injury. Panel (**A**) examines all-cause mortality and Panel (**B**) examines cardiovascular mortality, showing direct and indirect pathways through subclinical myocardial injury. Abbreviations: OR, odds ratio; HR, hazard ratio; SCMI, subclinical myocardial injury. Two mediation diagrams illustrating subclinical myocardial injury as an intermediary pathway between resting heart rate and mortality. Panel (**A**) shows five percent attenuation of the all-cause mortality hazard ratio from 1.20 to 1.19 per 10 beats per minute increase after adjusting for subclinical myocardial injury. Panel (**B**) shows 8.6 percent attenuation for cardiovascular mortality, from 1.16 to 1.14.

Adjustment for SCMI attenuated the association between elevated heart rate and mortality, suggesting that silent myocardial damage may partly account for the prognostic impact of elevated heart rate. The attenuation was modest for both outcomes, with SCMI accounting for approximately 5% of excess all-cause mortality risk and 8.6% of excess CV mortality risk, indicating that SCMI explains only a relatively small proportion of the heart rate–mortality association (Table 4, Figure 3).

### 3.5. Subgroup Analyses

The resting heart rate–SCMI association remained stable across every prespecified subgroup, with no significant effect modification by age, sex, race, hypertension, diabetes, or body mass index, underscoring the consistency of the finding across clinically relevant strata (Table 5).

## 4. Discussion

Among participants in NHANES III, a large population-based survey of U.S. adults with no baseline CVD, elevated resting heart rate was associated with increased prevalence of SCMI and with long-term mortality from all causes and CV causes. Importantly, SCMI modestly attenuated the relationship between elevated heart rate and mortality, even after adjustments were made for traditional CV risk factors. This suggests that silent myocardial damage may represent one potential intermediary pathway through which chronically elevated heart rate is associated with adverse outcomes, though the findings are consistent with partial mediation rather than establishing a definitive causal mechanism.

Consistent with our findings, prior work has repeatedly linked elevated resting heart rate to overt CV disease and adverse clinical outcomes; however, far fewer studies have examined whether heart rate relates to subclinical ischemic processes that precede clinical events [1,2]. Methods used to ascertain subclinical ischemic or atherosclerotic disease vary across cohorts, yet most population-based studies report prevalence estimates similar to ours. The prevalence of SCMI in our NHANES III cohort (24.3%) was comparable to previously reported rates in U.S. and European populations [8,9]. Similarly, studies that used coronary artery calcium (CAC) as an alternative index of subclinical disease, among them the Heinz Nixdorf Recall Study, the Multi-Ethnic Study of Atherosclerosis (MESA), and large non-Western Korean cohorts, have reported that a higher resting heart rate tracks with a heavier subclinical atherosclerotic burden [23,24,25]. Although these studies rely on CAC rather than ECG-based detection of myocardial injury, they collectively reinforce the concept that higher resting heart rate is consistently linked to early subclinical CV pathology across diverse populations.

Within our cohort, the odds of SCMI were more than doubled among participants with tachycardia, a relationship that held even after accounting for multiple CV risk factors. This aligns with physiologic mechanisms through which elevated resting heart rate may promote subclinical myocardial injury. Higher heart rate increases myocardial oxygen consumption while simultaneously shortening diastole, consequently decreasing coronary perfusion [5,26]. This mismatch between oxygen supply and demand may promote repetitive episodes of subclinical ischemia [27]. Over time, such repeated ischemic insults may result in cumulative myocardial damage detectable by ECG-based scoring systems. An elevated resting heart rate is also frequently regarded as a signature of autonomic dysregulation [28,29]. Reduced vagal tone has been linked to heart failure across several earlier studies, while persistent sympathetic activation has been tied to fibrotic remodeling of the myocardium, unfavorable ventricular changes, and heightened electrical instability [30,31]. Each of these processes may be associated with the development of SCMI. It should also be considered that elevated resting heart rate may itself represent a manifestation of occult autonomic dysfunction, systemic low-grade inflammation, or frailty rather than functioning as a direct causal mechanism for myocardial injury, and the observed associations may partly reflect shared upstream pathophysiology.

Our findings highlight that elevated resting heart rate may be a modifiable contributor to early myocardial injury rather than merely a secondary marker of CV stress. Although heart rate–lowering therapies such as β blockers, non-dihydropyridine calcium channel blockers, and ivabradine are widely used, their potential to slow early atherosclerotic and ischemic processes remains underleveraged [32,33]. Growing experimental and clinical insights suggest that heart rate reduction may favorably influence vascular biology, coronary perfusion, and myocardial oxygen balance; yet no population-based studies have evaluated whether lowering resting heart rate can prevent or attenuate subclinical myocardial injury through heart rate modulation [34,35]. This gap is particularly relevant given the prognostic importance of SCMI and its demonstrated attenuation of the heart-rate–mortality relationship in our cohort.

The association between elevated heart rate and CV mortality was modestly attenuated by SCMI (8.6%), indicating that SCMI did not fully explain the heart rate–CV mortality relationship, and that additional pathways beyond CIIS-detectable injury are likely operative. Some of these mechanisms include prothrombotic states or accelerated atherosclerosis, which are both independently associated with higher resting heart rate and adverse CV outcomes [25,36]. Given the modest attenuation by SCMI, further work is needed to identify the predominant non-ECG mechanisms linking elevated resting heart rate to CV mortality.

The smaller attenuation observed for all-cause mortality (5%) similarly indicates that SCMI explains only a limited component of the relationship between elevated resting heart rate and global mortality risk. Rather than acting as the dominant link in this relationship, subclinical myocardial damage may function as one marker of reduced physiologic reserve and potentiate a pro-inflammatory state with multisystem consequences [37,38]. This aligns with prior findings that elevated resting heart rate predicts non-CV death as strongly as CV death, suggesting that both heart rate and SCMI may serve as indices of global physiologic compromise [22]. Consistent with this interpretation, large cohort studies have linked subclinical myocardial injury to increased risk of death from diverse causes, not solely CV events [39,40]. Future studies pairing ECG infarction scores with cause-of-death may provide additional insight into which mortality categories are most strongly associated with SCMI.

## 5. Limitations

This study used data from NHANES III, conducted between 1988 and 1994. Changes in CV risk factor prevalence and treatment patterns since that time may limit applicability to contemporary populations. The cross-sectional assessment of heart rate and SCMI at baseline precludes determination of temporal sequence; while we hypothesize that elevated heart rate contributes to the development of SCMI, it is also possible that underlying cardiac abnormalities simultaneously elevate heart rate and predispose to subclinical injury. Resting heart rate was measured at a single time point, which may not fully capture an individual’s typical heart rate or heart rate variability over time. Lastly, because only 71 participants (1%) had tachycardia, the categorical estimates for this subgroup should be interpreted with caution given limited statistical power and wide confidence intervals. The continuous heart rate analyses, which leverage the full sample and avoid arbitrary categorization, provide more statistically robust and clinically informative estimates and should be considered the primary findings.

## 6. Conclusions

In this analysis of NHANES III participants, elevated resting heart rate was independently associated with increased odds of subclinical myocardial injury and with higher long-term risk of both all-cause and CV mortality. Importantly, SCMI modestly attenuated the association between elevated heart rate and mortality, suggesting that these findings are consistent with silent myocardial damage serving as one potential intermediary pathway through which chronically elevated heart rate is associated with adverse outcomes. Future research is needed to determine whether interventions that lower resting heart rate can reduce the incidence of SCMI and improve long-term survival.

## Figures and Tables

**Table 1:** Baseline Characteristics.

Characteristics: Mean (SD) */Numbers (%)	Total (*n* = 7152)	SCMI Absent (*n* = 5408)	SCMI Present (*n* = 1744)	*p*-Value
Age, years	59.0 (13.3)	57.7 (13.1)	63.0 (13.2)	<0.001
Female	3820 (53.4%)	2941 (54.4%)	879 (50.4%)	0.004
Race Ethnicity				
Non-Hispanic White	5208 (72.8%)	3930 (72.7%)	1278 (73.3%)	0.619
Non-Hispanic Black	1736 (24.3%)	1305 (24.1%)	431 (24.7%)	0.622
Other	208 (2.9%)	173 (3.2%)	35 (2.0%)	0.010
Education, ≥high school	3804 (53.1%)	2950 (54.5%)	854 (48.9%)	<0.001
Family income < 20 k	3233 (45.2%)	2348 (43.4%)	885 (50.7%)	<0.001
Current Smoker	1634 (22.8%)	1171 (21.7%)	463 (26.5%)	<0.001
Past Smoker	2242 (31.3%)	1680 (31.1%)	562 (32.2%)	0.364
Lipid Lowering Medications	214 (2.9%)	157 (2.9%)	57 (3.3%)	0.436
Antihypertensive Medications	1485 (20.7%)	1128 (20.9%)	357 (20.5%)	0.733
Body Mass Index, kg/m^2^	27.7 (5.51)	27.6 (5.34)	27.9 (5.99)	0.021
Serum Creatinine, mg/dL	1.11 (0.30)	1.10 (0.30)	1.10 (0.30)	<0.001
Systolic Blood Pressure, mm Hg	132 (19.6)	131 (19.2)	137 (20.2)	<0.001
Diastolic Blood Pressure, mm Hg	76.4 (10.2)	76.4 (9.98)	76.5 (10.8)	0.087
Total Cholesterol, mg/dL	217.3 (43.7)	216 (42.7)	220 (46.6)	0.001
Heart Rate, BPM	68.5 (11.5)	68.1 (10.0)	69.9 (12.9)	<0.001
Bradycardia	238 (3.3%)	193 (3.6%)	45 (2.6%)	<0.001
Normal Heart Rate	6843 (95.7%)	5176 (95.7%)	1667 (95.6%)	
Tachycardia	71 (1.0%)	39 (0.7%)	32 (1.8%)	
Thyroid disease	369 (5.2%)	274 (5.1%)	95 (5.4%)	0.532
Diabetes Mellitus	756 (10.6%)	503 (9.3%)	253 (14.5%)	<0.001
Hypertension	2430 (34.0%)	1732 (32.0%)	698 (40.0%)	<0.001
Dyslipidemia	1664 (23.3%)	1259 (23.3%)	405 (23.2%)	0.960

* Continuous data are shown as mean ± SD for normally distributed measures and as median (IQR) otherwise; categorical data are shown as n (%). For continuous variables, *p*-values derive from independent samples t-tests or Wilcoxon rank-sum tests as appropriate, and for categorical variables from chi-square tests. SCMI corresponds to a CIIS of 10 or higher on the standard 12-lead ECG. Family income < 20 k refers to a total annual family household income of less than $20,000. Bradycardia: ≤50 BPM; Normal Heart Rate: >50–<100 BPM; Tachycardia: ≥100 BPM. Abbreviations: BPM, beats per minute; SD, standard deviation; SCMI, subclinical myocardial injury.

**Table 2:** Association between Heart Rate Groups and Subclinical Myocardial Infarction.

HR Group	SCMI N (%)		Model 1		Model 2	
	Present	Absent	OR (95% CI)	*p*-Value	OR (95% CI)	*p*-Value
Normal HR	1667	5176	Ref	--	Ref	--
Bradycardia	45	193	0.650 (0.46–0.91)	0.012	0.66 (0.47–0.94)	0.022
Tachycardia	32	39	2.45 (1.52–3.96)	<0.001	2.34 (1.42–3.88)	<0.001
HR per 10 bpm increase	--		1.16 (1.11–1.22)	<0.001	1.14 (1.08–1.19)	<0.001

SCMI denotes a CIIS of 10 or higher on the standard 12-lead ECG. Model 1 included age, sex, race/ethnicity, years of education, and family income. Model 2 retained these covariates and added smoking, alcohol use, antihypertensive therapy, body mass index, systolic blood pressure, serum creatinine, dyslipidemia, diabetes, and thyroid disease. Bradycardia: ≤50 BPM; Normal heart rate: >50–<100 BPM; Tachycardia: ≥100 BPM. Abbreviations: BPM, beats per minute; OR, odds ratio; CI, confidence interval.

**Table 3:** Association Between Heart Rate and Cardiovascular Mortality.

Outcome	Participants/Events	Model 1		Model 2		Model 3	
		HR (95% CI)	*p*-Value	HR (95% CI)	*p*-Value	HR (95% CI)	*p*-Value
Normal HR	6843/889	Reference	--	Reference	--	Reference	--
Bradycardia	238/34	0.85 (0.60–1.19)	0.342	0.81 (0.57–1.17)	0.265	0.86 (0.60–1.23)	0.416
Tachycardia	71/10	2.38 (1.28–4.46)	0.006	2.05 (1.06–3.79)	0.033	1.96 (1.01–3.80)	0.046
HR per 10 bpm increase	--	1.16 (1.09–1.22)	<0.001	1.16 (1.10–1.23)	<0.001	1.14 (1.08–1.21)	<0.001

Model 1 included age, sex, race/ethnicity, years of education, and family income. Model 2 retained these covariates and added smoking, alcohol use, antihypertensive therapy, body mass index, systolic blood pressure, serum creatinine, dyslipidemia, diabetes, and thyroid disease. Model 3 further incorporated subclinical myocardial injury (SCMI), the inclusion of which attenuated the heart rate–CV mortality association by 8.6%. SCMI denotes a CIIS of 10 or higher on the standard 12-lead ECG. Bradycardia: ≤50 BPM; Normal heart rate: >50–<100 BPM; Tachycardia: ≥100 BPM. Abbreviations: BPM, beats per minute; HR, hazard ratio; CI, confidence interval.

**Table 4:** Association Between Heart Rate, SCMI and All-cause Mortality.

Outcome	Participants/Events	Model 1		Model 2		Model 3	
		HR (95% CI)	*p*-Value	HR (95% CI)	*p*-Value	HR (95% CI)	*p*-Value
Normal HR	6843/2186	Reference	--	Reference	--	Reference	--
Bradycardia	238/79	0.80 (0.64–1.01)	0.057	0.78 (0.61–0.99)	0.040	0.79 (0.62–1.01)	0.057
Tachycardia	71/46	3.54 (2.63–4.76)	<0.001	3.58 (2.63–4.88)	<0.001	3.45 (2.53–4.69)	<0.001
HR per 10 bpm increase	--	1.21 (1.17–1.25)	<0.001	1.20 (1.16–1.24)	<0.001	1.19 (1.15–1.24)	<0.001

Model 1 included age, sex, race/ethnicity, years of education, and family income. Model 2 retained these covariates and added smoking, alcohol use, antihypertensive therapy, body mass index, systolic blood pressure, serum creatinine, dyslipidemia, diabetes, and thyroid disease. Model 3 further incorporated subclinical myocardial injury (SCMI), the inclusion of which attenuated the heart rate–all-cause mortality association by 5%. SCMI denotes a CIIS of 10 or higher on the standard 12-lead ECG. Bradycardia: ≤50 BPM; Normal heart rate: >50–<100 BPM; Tachycardia: ≥100 BPM. Abbreviations: BPM, beats per minute; HR, hazard ratio; CI, confidence interval.

**Table 5:** Subgroup Analysis of the Association between Resting Heart Rate and Subclinical Myocardial Injury.

Subgroup		Odds Ratio (95% Confidence Interval) *^,‡^	Interaction *p*-Value ^‡^
Age	≤65	1.14 (1.07–1.21)	0.7030
	>65	1.16 (1.08–1.25)	
Gender	Men	1.20 (1.12–1.28)	0.956
	Women	1.10 (1.03–1.18)	
Race	White	1.19 (1.12–1.26)	0.131
	Black	1.08 (0.97–1.19)	
	Other	1.49 (0.99–2.21)	
Hypertension	Present	1.20 (1.13–1.26)	0.093
	Absent	1.09 (1.03–1.15)	
Diabetes	Present	1.16 (0.94–1.20)	0.1710
	Absent	1.09 (0.95–1.23)	
BMI	Normal	1.16 (1.08–1.25)	0.5610
	Overweight	1.14 (1.06–1.26)	
	Obese	1.11 (1.02–1.22)	

BMI classified as normal (18.5–24.9 kg/m^2^), overweight (25.0–29.9 kg/m^2^), or obese (≥30.0 kg/m^2^). * Odds ratios are expressed per 10 BPM increase in resting heart rate. ^‡^ The fully adjusted model comprised age, sex, race/ethnicity, years of education, family income, smoking, alcohol use, antihypertensive therapy, body mass index, systolic blood pressure, serum creatinine, dyslipidemia, diabetes, and thyroid disease. SCMI denotes a CIIS of 10 or higher on the standard 12-lead ECG. Abbreviations: BMI, body mass index; BPM, beats per minute; OR, odds ratio; CI, confidence interval.

## Data Availability

Data underlying this analysis are openly accessible through the National Health and Nutrition Examination Survey (NHANES), a program of the Centers for Disease Control and Prevention (CDC). The relevant laboratory, examination, and questionnaire files are available at https://www.cdc.gov/nchs/nhanes/ (accessed on 2 March 2026), and the corresponding mortality follow-up is obtainable via the National Center for Health Statistics (NCHS) Linked Mortality Files. This study generated no new primary data. The derived variables and analytic code supporting the findings can be provided by the corresponding author on reasonable request.

## Abbreviations

Acronym List	
BMI	Body mass index
bpm	Beats per minute
CIIS	Cardiac Infarction/Injury Score
CV	Cardiovascular
CVD	Cardiovascular disease
ECG	Electrocardiogram
HR	Hazards ratio
NHANES III	Third National Health and Nutrition Examination Survey
OR	Odds ratio
SCMI	Subclinical myocardial injury
